# Healthcare in transition in the Republic of Armenia: the evolution of emergency medical systems and directions forward

**DOI:** 10.1186/s12245-020-00328-3

**Published:** 2021-01-12

**Authors:** Sharon Chekijian, Nune Truzyan, Taguhi Stepanyan, Alexander Bazarchyan

**Affiliations:** 1 Department of Emergency Medicine, Yale University School of Medicine, 464 Congress Avenue, New Haven, CT 06519 USA; 2 Turpanjian School of Public Health, American University of Armenia, 40 Marshal Baghramyan Avenue, 0019 Yerevan, Armenia; 3 Yerevan Municipal Ambulance Services, 40 Dzorapi Street, 0015 Yerevan, Armenia; 4 National Institute of Health of Armenia, 49/4 Komitas Avenue, 375051 Yerevan, Armenia

**Keywords:** Armenia, Soviet Union, Transition, Emergency systems, Emergency medicine, Ambulance physician, Semashko, Ex-Soviet Republic

## Abstract

Armenia, an ex-Soviet Republic in transition since independence in 1991, has made remarkable strides in development. The crisis of prioritization that has plagued many post-Soviet republics in transition has meant differential growth in varied sectors in Armenia. Emergency systems is one of the sectors which is neglected in the current drive to modernize. The legacy of the Soviet Semashko system has left a void in specialized care including emergency care. This manuscript is a descriptive overview of the current state of emergency care in Armenia using in-depth key informant interviews and review of published and unpublished internal United States Agency for International Development (USAID) and Ministry of Health (MOH) documents as well as data from the Yerevan Municipal Ambulance Service and international agencies. The Republic of Artsakh is briefly discussed.

The development of emergency care systems is an extremely efficient way to provide care across many different conditions in many age groups. Conditions such as traumatic injuries, heart attacks, cardiac arrest, stroke, and respiratory failure are very time-dependent. Armenia has a decent emergency infrastructure in place and has the benefit of an educated and skilled physician workforce. The missing piece of the puzzle appears to be investment in graduate and post-graduate education in emergency care and development of hospital-based emergency care for stabilization of stroke, myocardial infarction, trauma, and sepsis as well as other acute conditions.

## Background

### The rising importance of emergency systems and emergency medical care on the global stage

With the shift of global disease burden to trauma and non-communicable diseases from infectious diseases such as tuberculosis, human immunodeficiency virus, and malaria, it has become clear that strictly funding vertical programs focused on a single disease discourages the development of strong healthcare delivery systems. This can be especially dangerous for acute life-threatening illness or in the setting of pandemics, mass casualties, or natural disasters [1]. Much of the growth in public health research funding has been also directed towards specific disease processes whereas little has been focused on emergency care research [2–4].

Emergency medicine (EM) was born in the United States (US) in the 1950s and 1960s with the widespread expansion of automobile travel, highway development, and advances in medicine that made life-saving intervention not only possible but expected. The first American emergency medicine resident was accepted in 1970 [5, 6]. Globally, EM is in various stages of development ranging from rudimentary care to hospital-based stabilization [7, 8]. EM focuses on providing timely care to victims of sudden and life-threatening injuries or emergencies to avoid death or disability [9]. The function of EM can be simplified into four main components: accessing emergency care, care in the community (cardiopulmonary resuscitation (CPR), first aid), care en route, and care upon arrival to receiving care at the healthcare facility. This is also known as the chain of survival.

The development of emergency care systems can be an extremely efficient way to provide care across many different conditions across the human life span. Conditions such as traumatic injuries, heart attacks, cardiac arrest, stroke, and respiratory failure are very time-dependent [9]. Moreover, outcomes are tied to delivering the patient to the most appropriate facility in a timely fashion. Properly organized emergency care systems save lives and multiply the impact of many other parts of the health system by providing a mechanism for early recognition of acute life-threatening conditions coupled with timely access to needed care [10]. In all these acute conditions, the key is the chain of survival. Many of the links in the chain of survival need strengthening and have been neglected in the current schema of priorities and funding [11, 12]

Prevention is still key to most public health interventions but it is now obvious that many deaths and long-term disabilities can be mitigated by a strong emergency care system. The World Bank estimates that “more than half the deaths and around 40% of the total burden of disease in low- and middle-income countries result from conditions that could be treated with pre-hospital and emergency care.” Many recent studies have ranked components of emergency care as among the most cost-effective public health interventions [10]. In fact, emergency care systems are therefore according to the World Health Organization (WHO) a “key mechanism for achieving a range of Sustainable Development Goal targets, including those on universal health coverage, road safety, maternal and child health, non-communicable diseases, infectious diseases, disasters and violence.” [10].

### Significance for Armenia

Armenia, an ex-Soviet Republic in transition since independence in 1991, has made remarkable strides in development. The crisis of prioritization that has plagued many post-Soviet republics in transition has meant differential growth in varied sectors in Armenia. Emergency systems is one of the sectors which is neglected in the current drive to modernize. A low-income country at the time of independence, Armenia is now classified by the World Bank as a high middle-income country [13]. Armenia is plagued by a high burden of non-communicable disease and unintentional injury seen in many low- and middle-income countries (LMICs). The reason for this inequity worldwide remains unclear but likely has to do with socioeconomic and modifiable risk factors, health lifestyle practices, and to some extent genetics. Rapid urbanization and over- and undernutrition also play a role as does lack of political momentum to enact impactful policy such as tobacco control and salt intake reduction. All these factors are compounded by weak primary care systems and low health service utilization [14–18]. Likewise, the increased burden of unintentional injury in LMICs due to road traffic crashes is multifactorial and the burden generally falls on the younger demographic [19–21]. Armenia’s high burden of cardiovascular risk factors and unintentional injury make improvement of emergency care for myocardial infarction, stroke, and road traffic crashes imperative.

## Data for the Republic of Armenia

After the collapse of the Soviet Union, the ensuing years saw a notable increase in mortality followed by a differential reversal depending on the particular dynamics at play in each ex-Soviet republic [17, 22–24]. Profiles for three disease entities in Armenia which are most pertinent to emergency care are discussed below.

### Ischemic heart disease, acute myocardial infarction, and cardiac arrest

Worldwide ischemic heart disease is the leading cause of death accounting for 31% of the major causes of death. Ischemic heart disease in Armenia is responsible for 37% of all deaths at a rate of 248.5 per 100,000. Armenia ranks 35 out of 175 countries in cardiac deaths [25]. Armenia falls in the region of Eastern Europe/Central Asia (EE/CA) with the highest per capita burden of cardiovascular disease [26]. The role of EMS in cardiac care is multifold. Once a call is placed to the dispatch center, instructions for cardiopulmonary resuscitation (CPR) can be given over the phone. Bystander CPR is known to improve rates of survival [27]. Immediate access to automated external defibrillators in the community is also key to survival for arrests due to shockable rhythms. Early detection of ST elevation myocardial infarction by EMS can minimize time to percutaneous coronary intervention (PCI) and ensure that the patient be transported to the correct center [28]. In outlying regions without PCI availability, early fibrinolytics could be considered [29]. In areas with hospital-based emergency medicine, prompt care is optimized by a STEMI alert and stabilization of the patient prior to PCI. In Armenia, all of these areas need strengthening in the current EMS schema.

#### Current care modalities

PCI is available in the capitol city at multiple locations including Nork Marash Medical Center, Erebuni, Astghik, Shengavit, University Hospital No 1, the Cardiology Institute (not active currently), Best Life, Aramyants, Armenia, and Sourp Grigor Luisavoritch. In the regions, interventional angiography is available in Goris, Gyumri, and Vanadzor, as well as in Stepanakert in Artsakh. By policy, the ambulance must transport any patient with a STEMI to the closest hospital with interventional capabilities. The cost of PCI for STEMI is covered by the Ministry of Health but only at certain approved centers.

### Acute stroke

Worldwide, the burden of deaths due to stroke is 10%. More than 75% of that burden falls on LMICs with the highest burden in East Asia followed by the EE/CA region [30, 31]. Acute stroke in Armenia is responsible for 9% of all deaths at a rate of 132.5 per 100,000 [25]. Armenia ranks 127 out of 175 countries in stroke deaths. The role of EMS in acute stroke is early activation of the system, stabilization of the airway, early recognition of stroke symptoms by standardized screening tool, and transport to the appropriate level stroke center. Currently, ambulance physicians in Armenia do not employ a standardized stroke scale. Although it is beyond the scope of this paper, there are many opportunities to advance stroke care by EMS optimization.

#### Current care modalities

Hospital-based stroke care in Armenia has advanced rapidly over the last 2 years, and intraarterial tissue plasminogen activator (TPA) and intraarterial clot retrieval are available at two comprehensive stroke centers in Yerevan. These interventions are not available in the regions and patients are often transported to Yerevan for further care if indicated. The cost of acute stroke care is covered by the Ministry of Health as of 2019.

### Road traffic crashes: fatalities and injuries

**Fig. 1:**
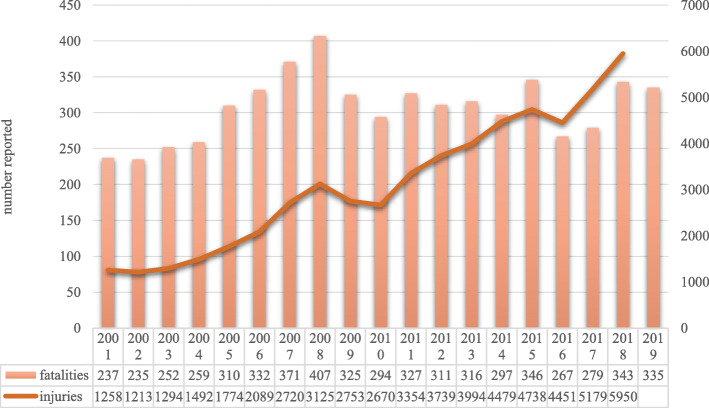
Reported number of road traffic fatalities and injuries in the Republic of Armenia [20, 33]

Deaths from road traffic injuries worldwide total approximately 1.35 million people per year. Ninety-three percent of these fatalities take place in LMICs. In 2016, the estimated road traffic death rate in Armenia (per 100,000) was 17.1. In neighboring countries, Azerbaijan was 8.7, Turkey was 12.3, Georgia was 15.3, and Iran was 20.5. As a reference, the United States is at 12.4, Canada is 5.8, and most European countries are in the 3–9 range with the higher rate in countries in the former eastern bloc (Poland, Latvia, Hungary). Russia is at 18.0 [21]. Barriers to road safety in Armenia include low safety belt usage, older vehicle fleets, poor road conditions, and lack of investment in proven engineering interventions [32] (Table 1; Fig. 1).

**Table 1:** Comparison of road traffic crash fatalities per 100,000 for 2016 [21]

Road traffic crash fatalities by region per 100,000 population (2016)	
**World**	**18.2**
Africa	26.6
Southeast Asia	20.7
Eastern Mediterranean	18.0
Western Pacific	16.9
Americas	15.6
Europe	9.0
**Former Soviet Republics**	**13.1**
Eurasia	18.0
Russia	18.0
Central Asia	15.4
Tajikistan	18.1
Kazakhstan	17.6
Kyrgyzstan	15.4
Turkmenistan	14.5
Uzbekistan	11.5
Southern Caucasus	13.7
Armenia	17.1
Georgia	15.3
Azerbaijan	8.7
Eastern Europe	10.7
Ukraine	13.7
Moldova^a^	9.7
Belarus	8.9
Baltic States	7.9
Latvia	9.3
Lithuania	8.0
Estonia	6.3

^a^Moldova data reported for 2018

### Considerations for disaster preparedness

In addition to the provision of acute care, disaster response and management are of the utmost importance to Armenia. This was highlighted by the Ministry of Health’s (MOH) “Republic of Armenia (ROA) Emergency Medical Systems Strategy Draft” which highlights that due to the Republic of Armenia’s geographical position and climate, the nation is subject to an increased probability of disasters. There is a high probability of natural disasters such as earthquakes, but Armenia is also at high risk for problems with an outdated nuclear power station, international terrorism, and war due to the lingering specter of the conflict in Artsakh [34]. A 2004 United Nations Development Programme (UNDP) report on reducing natural disaster risk revealed that during 1980–2000 Armenia averaged about 325 deaths per million inhabitants due to disasters. It is estimated that more than 80% of Armenians are at risk of exposure to catastrophic events [10, 35].

**Fig. 2:**
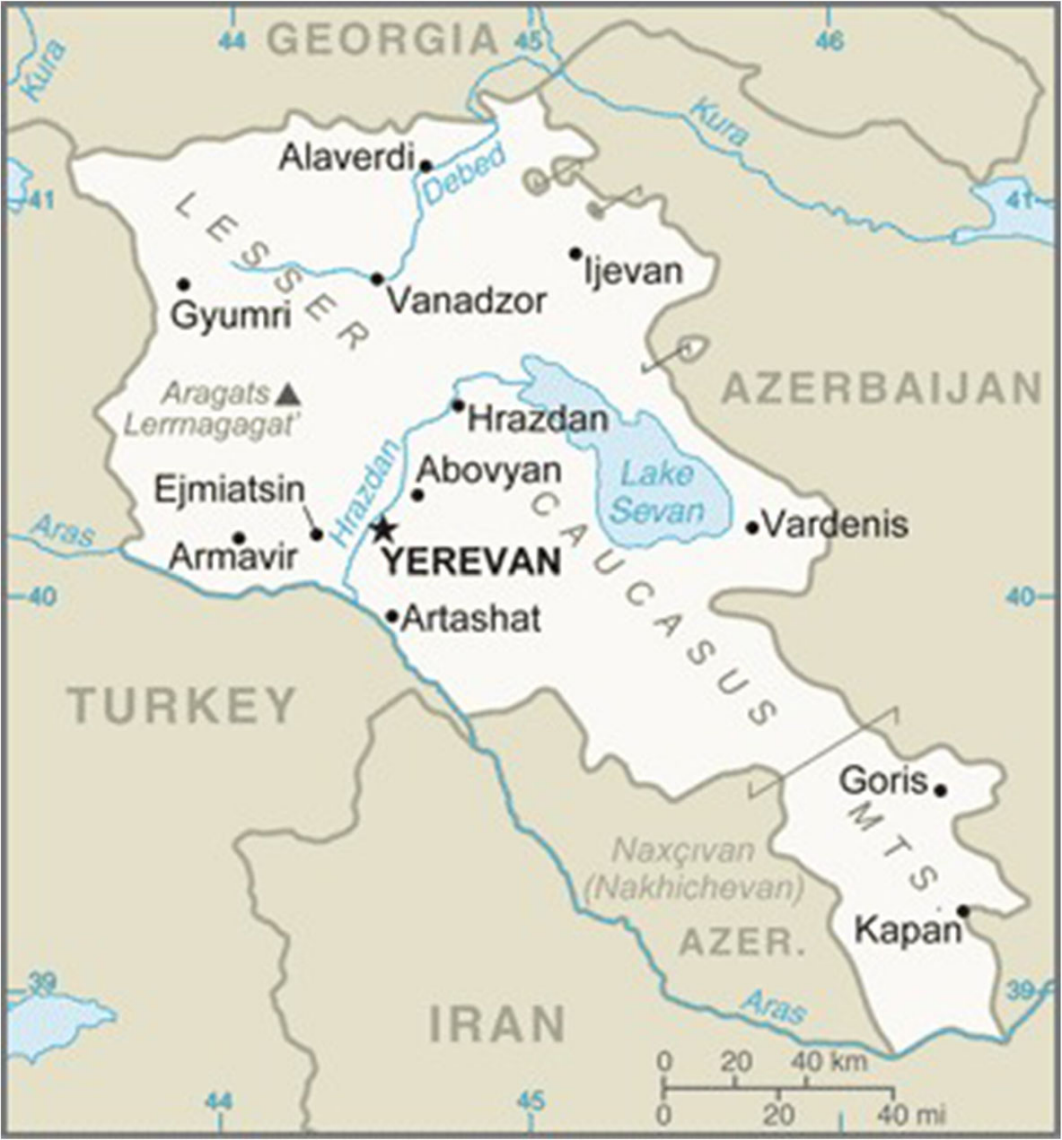
Republic of Armenia [36]

Besides meeting the everyday health needs of the population, a well-organized, prepared, and resilient emergency care system has the capacity to maintain essential acute care delivery throughout a mass casualty, limiting mortality and avoiding secondary mortality. Emergency care systems are an essential substrate for effective emergency response (Fig. 2).

## Current state of emergency care in the Republic of Armenia

### The transition

Following the earthquake of 1988, the subsequent fall of the Soviet Union, and the establishment of independent Armenia in 1991, the need to improve Armenian emergency medical systems became evident. The previous Soviet Semashko model which was based on a strict referral system from district doctor to central *rayon*, to municipal hospital, then *oblast*, and federal hospitals has largely broken down during the transition period. As a result, patients often self-refer to specialists, bypassing the referral system entirely. This can result in inaccurate diagnoses and delays in care especially in cases of acute conditions [37].

### Out-of-hospital and pre-hospital emergency care

#### Models of emergency care systems

Emergency medical systems (EMS) can be roughly divided into two models. These are the Anglo-American (scoop and run) and the Franco-German model (stay and stabilize) systems. Most EMS are an amalgamation of the two [38]. In the Anglo-American model, patients either present themselves or are brought to the hospital with protocol-driven pre-hospital interventions. Protocols become paramount as ambulances are staffed by paramedics and not by physicians. In the Franco-German model, physicians treat patients on scene or “out-of-hospital” and can make complex clinical judgments due to their training [38]. This results in most care being rendered on scene except for those patients who are critically ill. Armenia adheres to the Franco-German model with each ambulance team consisting of one physician, a nurse, and a driver.

The preponderance of emergency care is provided by the ambulance system, an inheritance from the Semashko system. The ambulance system is comprised of the Yerevan Municipal Ambulance Service (YMAS). The YMAS is under the municipal authority and has 35 ambulances distributed between the main station and its seven substations [35]. The municipal ambulance center is equipped with sophisticated communication equipment, including means for global positioning systems (GPS) monitoring of ambulance locations. It receives approximately 600 calls per day, of which 25% are considered true emergencies. The central dispatch center also acts to coordinate nationally to transfer patients usually from the regions to the capital city of Yerevan. There is capacity to coordinate EMS operations across regions if needed. Statistics are kept and reported to the Ministry of Health [35]. Utilization of EMS in Armenia is one of the highest in the world. Eighty percent of calls are treated on scene and 15–20% are transported to surrounding hospitals [39]. Approximately 40% of calls are “double calls” where a specialized ambulance is dispatched after initial evaluation for neurologic, cardiac, or psychiatric care. The time for “double calls” may add an additional 30–90 min to patient care making for an inefficient system for the most critical patients (Tables 2 and 3).

**Table 2:** Yerevan municipal ambulance service time metrics (2011) [39, 48]

Time metrics	Reported time range (min)^a^	Ideal time (min)
Time to dispatch	2–6	< 2
Time to arrival on scene	7–12	< 8
Time on scene	60–75	< 30
Transport time to hospital	10–15	10–15
Total time	79–108^b^	< 50

^a^Time intervals are tracked manually; time intervals not available for the regions

^b^Double calls may add 30–60 min per call

**Table 3:** Count and percent of ambulance activations in Armenia by location, disposition, type, and year

		2018		2019	
		*n*	(%)	*n*	(%)
**Armenia**	**Total ambulance activations**	**485,555**		**521,039**	
**Regions**	**Total ambulance activations**	**237,729**		**258,598**	
Unintentional and intentional injury activations in regions		12 226	(5.1%)	10 789	(4.2%)
**Yerevan**	**Total ambulance activations**	**247,826**		**262,441**	
Cardiac activations		54 423	(21.9%)	48 593	(18.5%)
Stroke activations		5 606	(2.2%)	5 930	(2.3%)
Unintentional and intentional injury activations		11 510	(4.6%)	12 802	(4.9%)
Unintentional and intentional injury activations with hospitalization		10 290	(89.4%)^a^	12 594	(98.3%)^a^
Total activations		54 137	(21.8%)	61 220	(23.3%)

^a^Unintentional and intentional injury activations with hospitalization percentages calculated out of total unintentional and intentional injury activations

The fleet of ambulances at YMAS and the regions is relatively updated after a gift of ambulances by the Chinese government in 2010. The ambulance fleet in Armenia consists of 85 ambulances. Additional ambulances are operated by hospitals and private service providers such as Erebuni, all of which can be used in a state of emergency. Helicopter services affiliated with Erebuni Hospital are also newly available as of the year 2019. The regional ambulance services operate under the “marzpetaran” or regional municipalities of which there are 10 in addition to Yerevan. Emergency service capacities outside the capital are described as limited. The population can access emergency services countrywide via the 1-03 number.

**Fig. 3:**
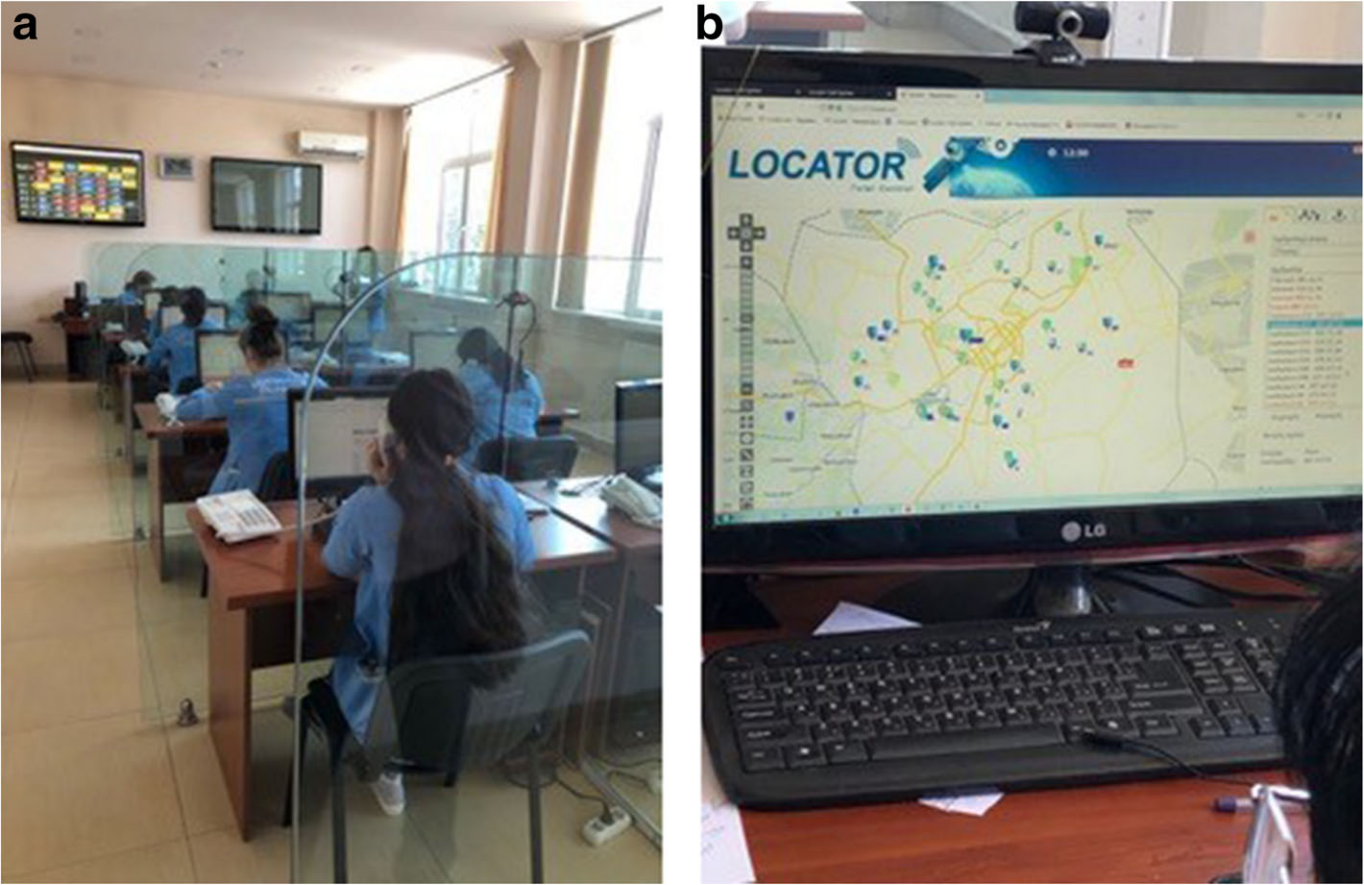
Yerevan central dispatch. GPS locator for calls (left) and 1-03 operators (right)

In 2009, the Armenian MOH prioritized reform of the emergency medical system. The Minister at the time identified professional development and expansion of human resource capabilities as a top priority. It is important to note that overall emergency and disaster response is not under the jurisdiction of the MOH but is instead under the Ministry of Emergency Situations established in 2008 and preceded by the “Headquarters of the Civil Defense” established in 1961 by Soviet authorities (Fig. 3).

#### Financing of emergency medical services

Ambulance services in Yerevan and in the regions were previously centralized under the jurisdiction of the MOH. This changed in the late 1990s. The Minister of Health at the time, Dr. Ara Babloyan, restructured regional ambulance services to fall under the direction and finances of the “marzpet” or regional governor who receives a payment from the budget office of the MOH yearly to cover the cost of ambulance calls. Medical direction is provided by each regional hospital. In Yerevan, ambulance services fall under the municipality. In Armenia’s second largest city of Gyumri, there is also a central station that falls under the direction and budget of the municipality. Emergency calls are fully subsidized by the budget office of the MOH. As of 2020 nationwide, there is no copayment made by the patient or family. Private ambulances, such as Erebuni, charge 15,000AMD (approximately $30 US) in the city regardless of the destination and by mileage for longer transport to or from the regions. Astghik hospital charges 10,000AMD (approximately $20 US) per call for transport to another hospital, but provides free transport to their center for treatment. Private ambulances are activated by a separate phone number outside of the 1-03 emergency number and maintained by the private hospitals.

## The Republic of Artsakh

**Fig. 4:**
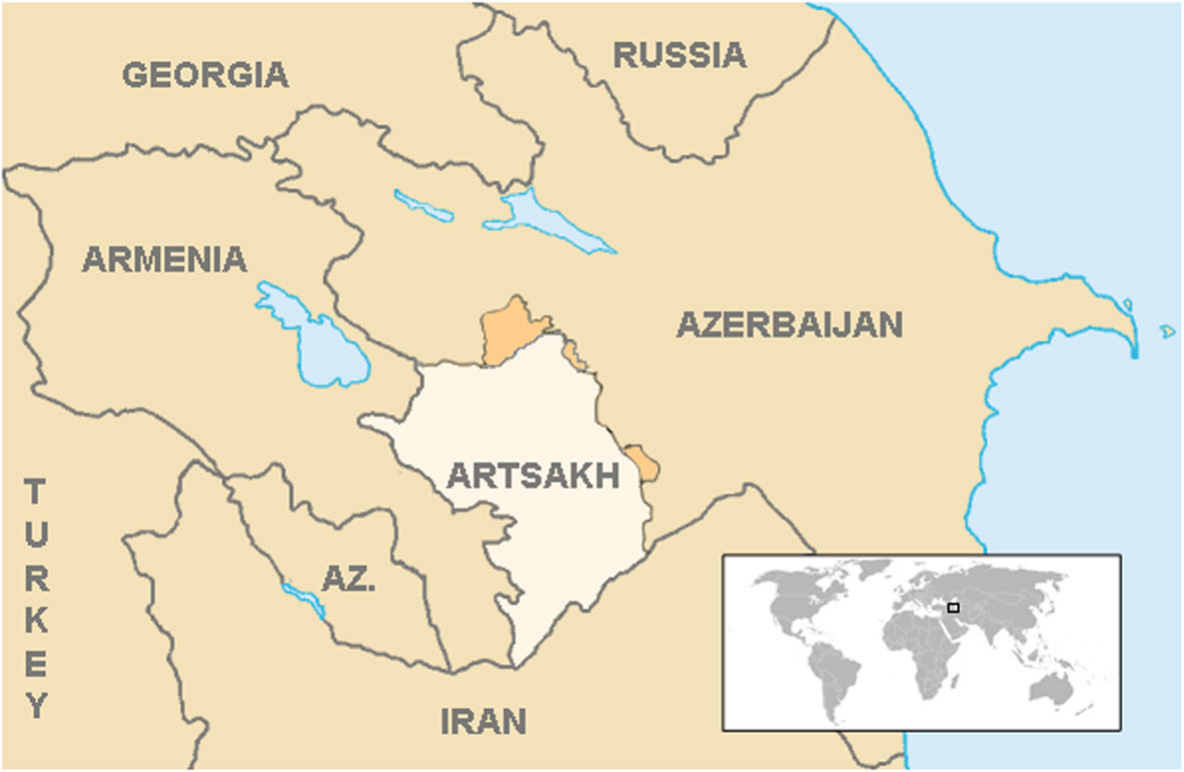
The Republic of Artsakh [40]

The ethnically Armenian, Republic of Artsakh to the west of the Republic of Armenia remains in a “frozen conflict” with Azerbaijan, and for reasons of national security, neither ambulance services nor the specifics of hospital-based emergency services will be discussed in detail except to note that they are more advanced than those currently found in Armenia. Artsakh has an autonomous Ministry of Health. There is no medical school in Artsakh. All graduate education and residency training for Artsakh physicians is undertaken in Yerevan or abroad. There is currently no emergency medicine-trained physician practicing in Artsakh. The Autonomous Republic remains unrecognized by the United Nations and is therefore not depicted in Figs. 1 and 4.

### Hospital-based emergency care

There is little hospital-based emergency care in the country with the exception of two private hospitals, Nairi and Erebuni. Erebuni Hospital currently has only an urgent care space but is building out a state-of-the-art Emergency Department with 24-h services and radiology. Nairi has a four-bed stabilization area called “Emergency Medical Care.” No data is available on the number or types of cases seen to date. It is staffed by a non-emergency-trained physician who can engage needed specialists and intensivists. The area is equipped for small procedures, intubations, and defibrillation. Once stabilized, the patient is immediately transferred to the intensive care unit.

There is no hospital-based emergency care outside of the capital city of Yerevan although there are “accepting rooms” or “endunaran” in all hospitals, including Yerevan, that are usually single unstaffed rooms without equipment where the patient is examined quickly prior to deciding on a disposition. There are no emergency-trained physicians in place; instead, the on-call doctor or nurse will call whichever specialist is thought to be needed and the patient will be sent to the appropriate ward whether medical or surgical. Obstetric hospitals are maintained separately. The absence of hospital-based emergency services leaves patients to fend for themselves when deciding to seek care for acute conditions such as trauma, stroke, and cardiac issues. Not all hospitals are capable of advanced care although some are well equipped to treat myocardial infarction or large vessel occlusion stroke with state-of-the-art intraarterial interventions. Much critical time can be lost in the process of self-referral especially when the patient arrives at a facility that does not have the ability to provide the needed critical or life-saving service.

### Development of emergency medical education

#### Emergency medical curriculum in graduate medical education

There are currently limited training opportunities in emergency medicine in the graduate medical curriculum. There are various courses in toxicology, public health, and epidemiology for undergraduate medical students at Yerevan State Medical University (YSMU). There are no emergency medicine rotations within the country. There is some coverage of disaster management for master’s degrees in public health including elements of public health in disasters, disaster medicine, and disaster management. All resident physicians are required to take a 5-week course on disaster management with modules on organization of medical response in emergency situations, minimization of casualties, surgery, medicine, obstetrics, and psychiatry in disasters.

#### Training of other specialists in emergency care

Ideally, emergency medicine will develop in Armenia as a specialty; however, there are advocates of preliminary efforts to embed the emergency medicine curriculum in post-graduate training for other specialties. This first step allows for awareness of acute conditions, accurate diagnosis by labs and diagnostic imaging, and stabilization and treatment. Ultimately, this is also beneficial to emergency specialists as their colleagues have already developed an awareness and a workflow for acute conditions and stabilization.

#### Training of nurses and other ancillary staff

Several efforts have been made to ameliorate nursing education in the country, notably a partnership between the University of California Los Angeles and Erebuni Hospital. These efforts would need to continue so that those working along-side newly formed emergency specialists are also working from the same diagnostic perspective and so that neither speed nor accuracy is lost in the process. Some of the work to stabilize patients is algorithmic. These algorithms must be shared. Medication administration which is largely the purview of the nurse is particular to critical care and is not inherently taught in nursing school. Likewise, a shared vision of the emergency model of medicine is essential.

#### Emergency medicine post-graduate training

The training of physicians to recognize acute life-threatening conditions requires a critical mass of physicians, nurses, and other paramedical staff who understand the principles of emergency care and who are evangelists for their inclusion in graduate and post-graduate curricula. Initially, this can occur within each specialty, but ultimately work needs to be done to advance the specialty of emergency medicine [1]. Currently, in the Republic of Armenia, EM is not taught as a discipline or administered as a post-graduate training program. With the assistance of USAID, there were early attempts in the late 1990s at emergency training courses for those in the field. Eventually, a 2-year residency program affiliated with the Yerevan State Medical University and instituted at St Grigor Luisavoritch Hospital which trained physicians for several years. To our knowledge, there have been no recent graduates of that program.

#### Current efforts to develop post-graduate medical education

There are currently 105 full-time and 197 part-time physicians working in the ambulance system in the capital city of Yerevan [39]. None is residency trained in emergency medicine. Most have received post-graduate training in another specialty such as cardiology, obstetrics, pediatrics, or general medicine. The National Institute of Health is currently working to establish new post-graduate programs in emergency medicine. This effort will start by working with the municipal ambulance system to voluntarily retrain already practicing doctors employed by the ambulance services in order to standardize their education and bolster their depth of knowledge. The curriculum would be a 1-year certificate or fellowship curriculum to bolster the knowledge of doctors already working for the municipal ambulance services. Ideally, in the future, this certification will be a requirement of employment. Natural leaders will be chosen from this group with enhanced training so that they can advance and become lead faculty in a new 3-year post-graduate residency program in emergency medicine [41–43].

## Emergency systems data collection and evaluation

According to the WHO assessment of health systems crisis preparedness, there is no systematic research on emergency systems currently taking place in Armenia. There is no mechanism within the MOH to prioritize the scientific research agenda, nor has it been budgeted [35]. There is, however, a large amount to data being collected by the YMAS and the regional ambulance services, as well as the police and individual hospitals on admission. The uniform collection and analysis of data should be prioritized, examined, and organized to direct future efforts and policy [44–47].

In addition to the development of graduate and post-graduate education and expansion of hospital-based emergency care, there are several unaddressed items suggested by the WHO as important to any country’s development of EMS (Table 1). Given the ongoing “frozen conflict” with Azerbaijan and for reasons of national security, it is reasonable to expect that not all of these items will be investigated and publicly discussed. This raises the question of the universal applicability of these items in other conflict zones (Tables 4).

**Table 4:**
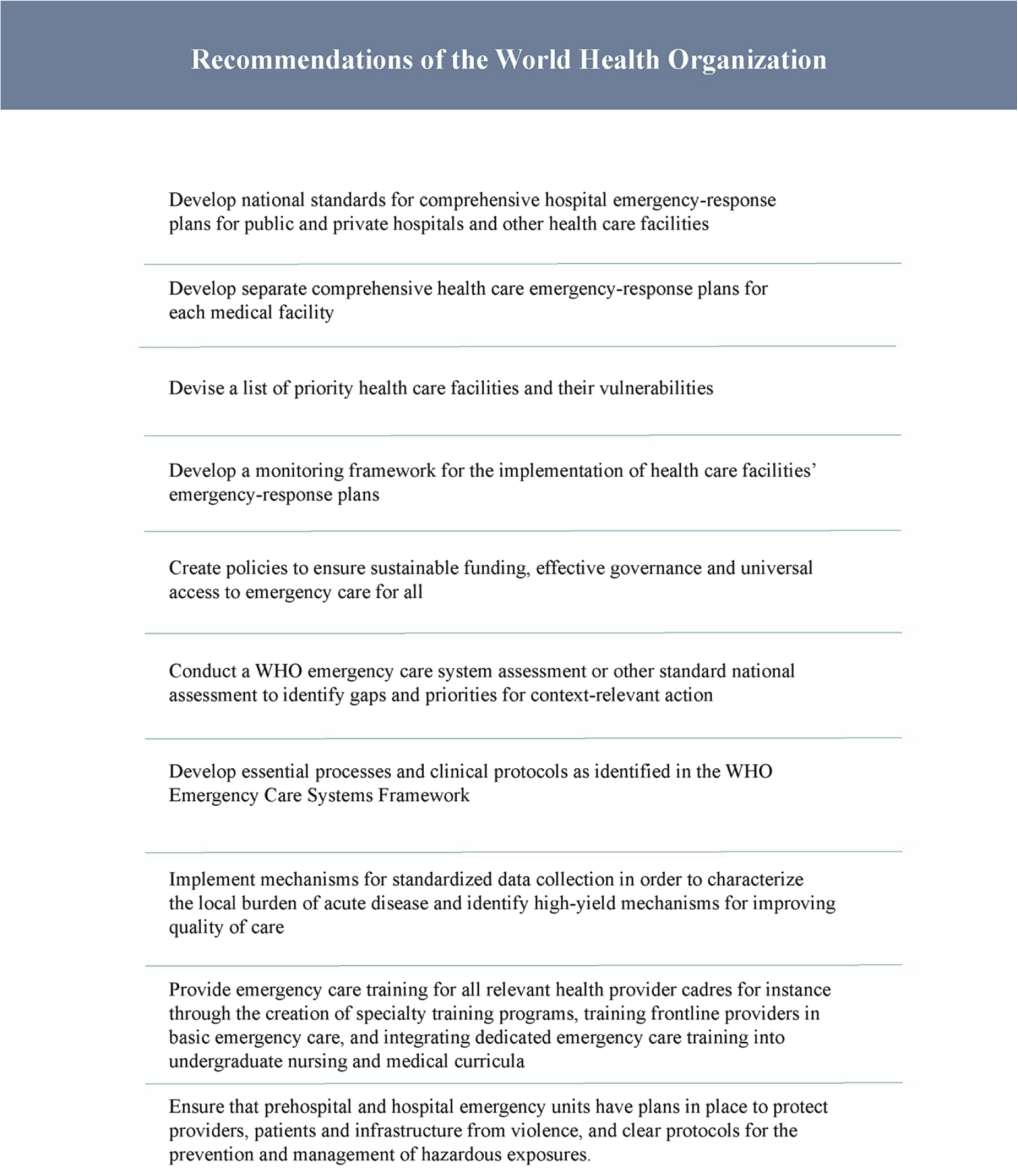
WHO recommendations [49, 50]

## Recommendations and conclusions

The period of post-Soviet transition has been one defined by a crisis of prioritization. Emergency care has remained fairly undeveloped, yet it has the potential to catalyze the entirety of healthcare. The importance of emergency care systems development cannot be underestimated. Much progress has been made in cardiac and stroke care in Armenia and up-to-date advancements are available. An investment in emergency systems can exponentially boost cardiac, stroke, and trauma care programs and thus the health of a nation. There are many opportunities to align emergency care with Armenia’s state-of-the-art offerings including integration of EMS within the cardiac and stroke protocols to bolster every link in the chain of survival. Armenian finds itself in a risky geopolitical region. Emergency medical systems can mitigate this risk and provide a level of confidence necessary for repatriation and for further economic development of sectors such as tourism and technology. Armenia has the advantage of a robust infrastructure and a political will on the part of the National Institute of Health (NIH) and the MOH to improve emergency care. However, much work remains to be done, especially in the area of development of human capital through education. In summary, strengthening EMS in cardiac and stroke chains of survival, improving current emergency services through education and training of existing staff, developing graduate and post-graduate training, and expanding regional and hospital-based emergency care are tantamount to EMS development and quality improvement in the Republic of Armenia.

## Data Availability

Not applicable
